# Back pain and health status in patients with clinically diagnosed ankylosing spondylitis, psoriatic arthritis and other spondyloarthritis: a cross-sectional population-based study

**DOI:** 10.1186/s12891-016-0960-8

**Published:** 2016-02-27

**Authors:** Ulf Lindström, Ann Bremander, Emma Haglund, Stefan Bergman, Ingemar F. Petersson, Lennart T. H. Jacobsson

**Affiliations:** Department of Rheumatology and Inflammation Research, Institute of Medicine, Sahlgrenska Academy, University of Gothenburg, Guldhedsgatan 10A, 405 30 Gothenburg, Sweden; Department of Clinical Sciences, Section of Rheumatology, Lund University, Lund, Sweden; School of Business, Engineering and Science, Halmstad University, Halmstad, Sweden; Spenshult Research and Development Center, Halmstad, Sweden; Primary Health Care Unit, Department of Public Health and Community Medicine, Institute of Medicine, Sahlgrenska Academy, University of Gothenburg, Gothenburg, Sweden; Department of Clinical Sciences, Section of Orthopedics, Lund University, Lund, Sweden

**Keywords:** Epidemiology, Spondyloarthritis, Psoriatic arthritis, Ankylosing spondylitis, PROMs

## Abstract

**Background:**

In the broader spectrum of back pain, inflammatory back pain (IBP) is a symptom that may indicate axial spondyloarthritis (SpA). The objectives of this study were to determine the frequency of current IBP, as a hallmark sign of possible axial SpA, in patients with ankylosing spondylitis (AS), psoriatic arthritis (PsA) and other SpA and to compare self-reported health between the groups with current IBP.

**Methods:**

Five-thousand seven hundred seventy one patients identified in the regional healthcare register of the most southern county of Sweden, diagnosed at least once by a physician (based on ICD-codes) with any type of SpA in 2003–2007, were sent a postal survey in 2009. Patients with current IBP were identified, based on self-reported back pain ≥3 months in the preceding year and fulfilling the Berlin criteria for IBP. The frequencies of IBP in AS, PsA and other SpA (including the remaining subgroups of SpA) were determined, and the groups were compared with regard to patient reported outcome measures (PROMs).

**Results:**

The frequency and proportion of patients with current IBP in AS, PsA and other SpA were 319 (43 %), 409 (31 %) and 282 (39 %) respectively, within the responders to the survey (*N* = 2785). The proportion was statistically higher in AS, compared to PsA (*p* < 0.001), but not for AS compared to other SpA (*p* = 0.112). PsA and other SpA, with current IBP, had similar (BASFI, EQ-5D, patients global assessment, fatigue, spinal pain) or worse (BASDAI) PROMs, compared to AS with current IBP. PsA with current IBP received pharmacological, anti-rheumatic, treatment more frequently than AS with current IBP, while AS and other SpA received treatment to a similar degree.

**Conclusion:**

The proportion of patients with current IBP was substantial in all three groups and health reports in the non-AS groups were similar or worse compared to the AS group supporting the severity of IBP in these non-AS SpA groups. These findings may indicate a room for improvement concerning detection of axial disease within different subtypes of non-AS SpA, and possibly also for treatment.

**Electronic supplementary material:**

The online version of this article (doi:10.1186/s12891-016-0960-8) contains supplementary material, which is available to authorized users.

## Background

The spondyloarthritis (SpA) group includes a number of similar inflammatory diseases, with varying degrees of inflammation in peripheral joints, entheses, spine, gut, skin, eyes and other organs. In a clinical setting SpA-disease is often categorized through its most prominent feature, e.g. psoriatic arthritis (PsA), SpA associated with inflammatory bowel disease (IBD), reactive arthritis (ReA) or axial SpA. In axial SpA the most prominent feature is chronic back pain, usually accompanied with symptoms of inflammatory back pain (IBP) and often with signs of inflammation in the sacroiliac joints and spine [1, 2]. Axial SpA having resulted in radiographically detectible sacroilitis is clinically diagnosed as ankylosing spondylitis (AS) [3], while axial SpA without radiographic sacroilitis may be classified as non-radiographic axial-SpA, according to the “Assessment of SpondyloArthritis” (ASAS) criteria for axial SpA[1].AS is known to be more common in men and non-radiographic axial SpA in women, and the progression rate from non-radiographic axial SpA to AS is considered to be around 10–12 % over 2 years [1].

The most common presentation of axial SpA is IBP [2], wherefore this is one of the key symptoms that are asked for in a clinical setting, when trying to identify patients with axial SpA. However, neither the modified New York criteria for AS [4] nor the current ASAS criteria for axial SpA[5] includes past or present IBP as a compulsory feature. In fact, both the sensitivity and the specificity of IBP for axial SpA, classified according to the ASAS-criteria in the setting of established axial SpA or chronic back pain, is 70–80 % [6, 7], illustrating the fact that not all patients with axial SpA have IBP and that axial disease activity may vary over time [8].

The frequency and disease activity of axial disease in other subtypes of SpA, where chronic back pain is not the predominant symptom, i.e. patients not referred to a rheumatologist due to back pain or patients with a predominantly peripheral disease, is less studied. In one study, comparisons between patients with AS, PsA and SpA associated with IBD (all with radiographic sacroilitis) indicated no significant differences with regard to axial disease activity[9]. This comparison was however limited by excluding all forms of non-radiographic axial disease and by only focusing on patients with typical radiographic findings of sacroilitis. In patients with PsA attending a rheumatology clinic the frequency of IBP has been described to be as high as 49 % [10], and one recent study demonstrated a high frequency of IBP among young patients with cutaneous psoriasis (17 %) [11], which may indicate that axial disease is more common in psoriasis than previously thought. In conclusion, there is relatively little knowledge of how common symptoms of IBP are among different subtypes of SpA and how this is reflected in patient-perceived disease activity.

Our objectives in this cross-sectional, population based, study was to firstly, assess the proportion of patients with IBP within different subtypes of SpA, and secondly, to compare self-reported disease activity between the groups with IBP, in order to explore possible differences with regard to self-perceived severity. The study is based on a well-established and validated cohort of clinically diagnosed SpA patients, the SpAScania cohort.

## Methods

### Setting

This cross-sectional study was performed in the county of Skåne in southern Sweden, with a population of 1.2 million. Population-based healthcare registers were used to identify cases with SpA in Skåne, to whom a postal survey was sent in 2009.

### Data sources

The registers used in this study were the Skåne Health Care Register (SHCR) [12] and the Prescribed Drugs Register. All data in the registers were linked to the patients’ personal identification numbers (PIN), which are unique identifiers given to all residents in Sweden. The PIN allows the information on individuals in different registers to be cross-linked [13]. Additional file 1: Table S1 presents the ICD-10 (International Classification of Diseases) and ATC-codes (Anatomical Therapeutic Chemical codes) [14] that were used in the study.

The SHCR contains information from every patient visit to healthcare providers included in the national reimbursement system (the majority). Outpatient, inpatient, primary, and specialized care providers are all included. The data collected include disease codes, according to the Swedish version of the ICD-10 [15] (up to eight codes/visit), dates of visit/admission/discharge, and other administrative data.

The Prescribed Drugs Register collects information on all drugs dispensed in Sweden since July 2005: date of prescription/dispensation and the prescribed dose. The drugs are classified according to the ATC classification system [14].

### Ethical approval

The Regional Ethical Review Board at Lund University, Sweden, approved the study (301/2007, 406/2008). Informed consent was obtained in compliance with the Declaration of Helsinki.

### Study population

All patients in the SHCR who were ≥15 years of age and had a healthcare visit to a physician between January 2003 and December 2007 that resulted in an ICD-10 code that indicated a SpA diagnosis were identified (*N* = 6799, the SpAScania cohort). The diagnoses used were those for AS (ICD-10 codes M45 and M08.1), undifferentiated SpA/Sacroilitis (ICD-10 codes M46.0, M46.1, M46.8 and M46.9), IBD-associated arthritis (ICD-10 codes M07.4 and M07.5), PsA (ICD-10 codes L40.5 and M07.0-3), and ReA (ICD-10 codes M02.0-2, M02.8–9, M01.2 and M03.2). The validity of the SpA-diagnoses in the SpAScania cohort have previously been evaluated and found to be high [12, 16].

In 2009, all of the subjects identified in the procedure above, ≥18 years old, and still living in the county, were invited to participate in a postal survey (*N* = 5771). For the same subjects, the ICD-10 codes for the common SpA-related disease manifestations (IBD, psoriasis, and anterior uveitis), registered at a visit to a physician in primary or secondary care between 1998 and 2009 were extracted from the SHCR (see Additional file 1: Table S1). The same subjects were also linked to the Prescribed Drugs Register and the ATC-codes for the following SpA-related drugs (available for 2005–2009) were collected: the synthetic disease-modifying anti-rheumatic drugs (sDMARDs): methotrexate and sulphasalazine; and the tumor necrosis factor alpha inhibitors (TNFi): etanercept and adalimumab. Infliximab was not included as this is normally not collected at a pharmacy. The survey included a number of well-validated generic and disease-specific patient-reported outcome measures (PROMs): spinal pain, the patient’s global assessment of back disease and fatigue, the Bath Ankylosing Spondylitis Activity Index (BASDAI), the Bath Ankylosing Spondylitis Functional Index (BASFI) (all measured 0–10, best to worst, on a numerical rating scale (NRS)) and the European Quality of Life-5 Dimensions index (EQ-5D; higher score = better health)[17]. Included were also questions about frequency and duration of back pain; the different features of inflammatory back pain (IBP); family history of SpA; current pharmacological treatment and history of psoriasis and IBD.

### Case definitions

AS was defined as having received an ICD-10 code for AS at ≥1 physician visit during the 2003–2007 study period. AS is per definition an axial disease and the ICD-codes for AS in the SHCR have been validated previously [12]. All the remaining patients in the cohort had received another clinical diagnosis of SpA, and never a diagnosis of AS, during the 2003–2007 study period. This cohort was further divided into psoriatic arthritis (PsA) (defined as having ever either received a diagnosis for psoriasis in addition to a diagnosis of SpA, and/or psoriatic arthritis) and “other-SpA” (having never received neither a diagnosis of AS nor PsA). Among the responders to the survey we identified all cases, within the AS, PsA and “other-SpA” groups, with current IBP, based on self-reported back pain (pain in the back or buttocks) for ≥3 months within the last 12 months and fulfilling the Berlin criteria for IBP. The Berlin criteria for IBP (rather than the ASAS criteria) were used for identification of IBP since they may have a higher specificity and may also perform better in PsA [6, 7, 11].

### Reliability analysis

To compare the concordance (and thus indirectly the reliability) of data collected by the survey and the registers (SHCR and the Prescribed Drugs Register), kappa-values for variables that could be identified in both sources were calculated. The variables included were current treatment with methotrexate, sulphasalazine, etanercept, or adalimumab, and existence of psoriasis or IBD.

### Statistical methods

The frequency and proportion of cases with current IBP was determined for each of the three groups, AS, PsA and other-SpA, as a total and stratified by sex and 15-year interval age groups. Between group comparisons were performed using Fisher’s exact test.

The three groups of AS, PsA and other-SpA, with current IBP, were compared in terms of levels of PROMs and frequencies of SpA-related disease manifestations, and pharmacological treatment by using independent-sample t-tests and Fisher’s exact test. Due to the multiple testing performed on the same data set, a Bonferroni correction was performed to counteract the risk of a type I error. More specifically comparisons of frequency of PROMs and pharmacological treatment were done using a *p*-value with the cut-off of 0.00125, obtained through a Bonferroni correction based on a statistical significance of 0.05 and the number of comparisons being 40.

SAS version 9.3 for Windows was used for the aggregation of data and SPSS version 21 for Windows was used for statistical analyses.

## Results

### The SpAScania cohort

**Fig. 1:**
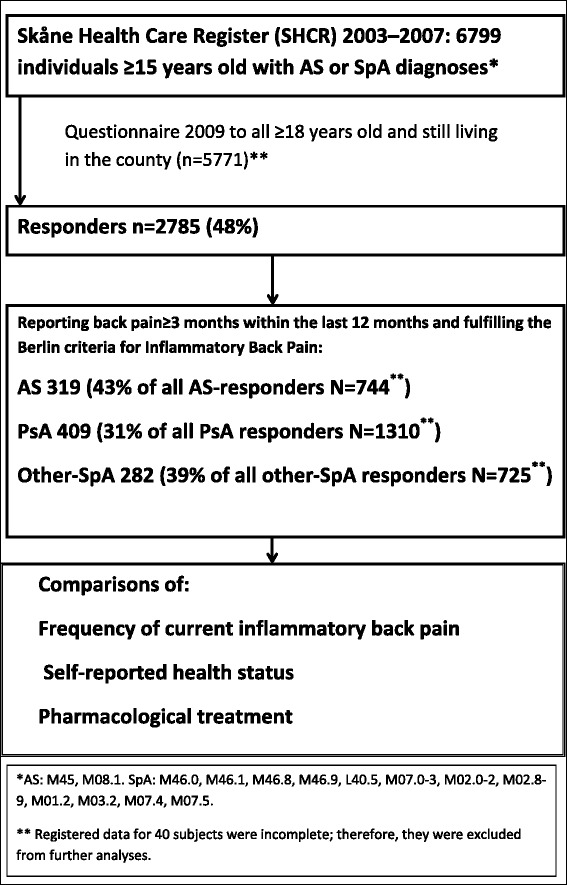
Flow chart depicting the selection of the SpAScania cohort. Legend: The figure shows the identification of patients with ankylosing spondylitis (AS), psoriatic arthritis (PsA) and other spondyloarthritis (SpA) in the Skåne Health Care Register, the proportions reporting current inflammatory back pain (back pain ≥ 3 months within the last 12 months and fulfilling the Berlin criteria for IBP), the response to the questionnaire, and the subsequent analyses

In total, 5771 individuals received ICD-10 codes for AS, PsA and other SpA in 2003–2007, were ≥18 years old, and were living in the county at the time of the survey (2009). Their mean age was 54.7 years and 53 % were women. Of these, 1423 (41 % women) had an AS diagnosis and 2280 (57 % women) had PsA (had ever received a diagnosis of PsA or alternatively any other non-AS SpA diagnosis in combination with a diagnosis of psoriasis) and 2028 (57 % women) had another SpA diagnosis. Registered data for six responders and 32 non-responders were incomplete and data for two additional subjects were missing when assembling the data-sets, these were excluded from further analyses. Complete responses to the questionnaire were received from 48 % (*n* = 2785) of recipients. A flow chart showing the analysis steps is presented in Fig. 1.

Of the 2785 responders 319 (43 % of the AS group, 43 % women) had AS with symptoms of current IBP (reported back pain ≥3 months within the last 12 months and fulfilled the Berlin criteria for IBP), 409 (31 % of the PsA group, 68 % women) had PsA with current IBP and 282 (39 % of the other-SpA group, 69 % women) had other-SpA with current IBP (Table 1) The differences in the proportions with current IBP was statistically significant for AS and other-SpA compared to PsA but not for AS compared to other-SpA. The occurrence of IBP was highest in the age-group 45–59, where the difference between AS and PsA was also most pronounced, and decreased after the age of 60. The proportions with IBP were, furthermore, overall higher in women compared to men, although it reached statistical significance only in other-SpA and PsA, but not in AS. (Table 1).

**Table 1:** Frequency and proportion with current inflammatory back pain among AS, PsA and other-SpA. Legend: For each group only data for responders to the postal survey (*N* = 2785) are included, stratified first on sex and then on age-groups. Registered data for 6 responders were incomplete and are thus excluded from the study

	Ankylosing Spondylitis (AS) *N* = 744		Psoriatic arthritis (PsA) *N* = 1310		*p*-value AS vs PsA	Other Spondyloarthritis (SpA) *N* = 725		*p*-value AS vs SpA	*p*-value PsA vs SpA
Men (%)	451 (61)		552 (42)		<0.001	286 (39)		<0.001	0.240
Current IBP^a^ (%)	319 (43)		409 (31)^b^		<0.001	282 (39)^c^		0.112	0.001
Sex	Women *N* = 293	Men *N* = 451	Women *N* = 758	Men *N* = 552		Women *N* = 439	Men *N* = 286		
Current IBP *N*(%)	137 (47)	182 (40)	280 (37)	129 (23)		195 (44)	87 (30)		
*p*-value men vs women	0.111		<0.001			<0.001			
Age groups	Total	Current IBP (%)	Total	Current IBP (%)		Total	Current IBP (%)		
15–29	42	12 (29)	50	9 (18)	0.319	72	29 (40)	0.231	0.010
30–44	190	82 (43)	241	73 (30)	0.006	185	74 (40)	0.600	0.040
45–59	243	126 (52)	463	172 (37)	<0.001	222	96 (43)	0.077	0.133
60–74	226	86 (38)	464	133 (29)	0.015	198	64 (32)	0.224	0.354
>75	43	13 (30)	92	22 (24)	0.303	48	19 (40)	0.519	0.077

All *p*-values are based on Fisher’s exact test

^a^IBP = inflammatory back pain, according to the Berlin criteria

^b^Proportion of IBP in psoriatic arthritis compared to ankylosing spondylitis *p* < 0.001

^c^Proportion of IBP in other-spondyloarthritis compared to ankylosing spondylitis *p* = 0.112

### PROMs and pharmacological treatment

All PROMs (spinal pain, fatigue, patients global, BASFI and EQ-5D) were similar in the three groups, apart from BASDAI which was significantly higher in the PsA group compared to the AS-group. Using a significance level of 0.05 (without Bonferroni correction) resulted in additional significantly worse scores for PsA compared to AS for fatigue, and for other-SpA compared to AS for spinal pain and BASDAI. The mean differences between AS, PsA and other-SpA in PROMs were however in absolute numbers small and within a range that is normally not considered clinically significant [18, 19]. Table 2 summarizes the demographics, SpA features, PROMs, and pharmacological treatment of the three groups with current IBP.

**Table 2:** Spondyloarthritis features, pharmacological treatment, and PROMs in AS, PsA or other-SpA, with IBP

	AS with current IBP^a^ *N* = 319	PsA with current IBP^a^ *N* = 409	*p*-value*	Other-SpA with current IBP^a^ *N* = 282	*p*-value**	*p*-value***
Demographic						
Age 2009, mean (sd)^b^	54 (13)	57 (13)		53 (15)		
Sex, N men (%)^b^	182 (57)	129 (32)	<0.001	87 (31)	<0.001	0.860
SpA-related disease, *N* (%)						
Uveitis^b^	63 (20)	9 (2)	<0.001	37 (13)	<0.001	<0.001
Psoriasis^b^	19 (6)	407 (100)	<0.001	0		
Inflammatory bowel disease^b^	25 (8)	21 (5)	0.901	36 (13)	0.706	0.001
PROMS, mean (95 % Cl)						
NRS-spinal pain^c^	5.9 (5.6−6.1)	6.1 (5.9−6.4)	0.068	6.2 (6.0−6.5)	0.037	0.688
NRS-fatigue^c^	5.8 (5.6−6.1)	6.2 (5.9−6.4)	0.044	6.1 (5.8−6.4)	0.117	0.547
NRS-patients global^c,d^	5.2 (4.9−5.4)	5.4 (5.2−5.6)	0.188	5.5 (5.2−5.7)	0.087	0.850
BASDAI^c^	5.1 (4.8−5.3)	5.7 (5.5−5.8)	<0.001	5.5 (5.3−5.8)	0.006	0.530
BASFI^c^	4.4 (4.2−4.7)	4.8 (4.5−5.0)	0.093	4.4 (4.1−4.7)	0.973	0.093
EQ-5D^c^	0.68 (0.66−0.70)	0.65 (0.63−0.67)	0.062	0.67 (0.64−0.69)	0.362	0.421
Treatment, *n* (%)						
Etanercept^b^	27 (9)	54 (13)	0.044	15 (5)	0.150	0.001
Adalimumab^b^	12 (4)	32 (8)	0.027	9 (3)	0.825	0.013
Methotrexate^b^	37 (12)	151 (37)	<0.001	33 (12)	1.000	<0.001
Sulphasalazine^b^	28 (9)	14 (3)	0.003	24 (9)	1.000	0.006

*Abbreviations*: *AS* ankylosing spondylitis, *IBD* inflammatory bowel disease, *NRS* numerical rating scale 1–10, *BASDAI* bath ankylosing spondylitis activity index, *BASFI* bath ankylosing spondylitis functional index, *PROMs* patient-reported outcome measures, *IBP* inflammatory back pain

Based on 2785 spondyloarthritis patients in the Skåne Health Care Register who responded to a questionnaire, registered data for 6 responders were incomplete and was thus excluded

*^, ^**AS with current IBP vs. PsA and other-SpA with current IBP, respectively

***PsA with current IBP vs. other-SpA with current IBP

^a^Current IBP: ≥3 months of back pain in the preceding year and fulfilling the Berlin criteria for inflammatory back pain (IBP)

^b^Data from the register (SpA-related disease 1998–2009; treatment 2005–2009)

^c^Data from the survey

^d^Patient’s global assessment of back disease

PsA, with current IBP, used methotrexate more frequently than the AS and other-SpA groups with current IBP and etanercept more frequently than the other-SpA group. No differences in treatment were observed between AS and other-SpA with current IBP.

Stratifying the groups by sex revealed overall worse results for all PROMs in women compared to men, but similar results with regard to differences in men and women between AS, PsA and other-SpA as those seen in the overall comparison (Additional file 1: Table S2).

### Responders vs. non-responders and reliability analysis

Table 3 shows the differences between the responders and non-responders in terms of age, gender, SpA diagnosis, and pharmacological treatment. The non-responders differed significantly from the responders in that they were younger, and more likely to have been given a diagnosis of ReA. Non-responders also were slightly less likely to have been given diagnoses of AS, undifferentiated SpA, and PsA. These differences in diagnoses probably explain why the non-responders also had lower frequencies of use of sDMARDs and TNF-inhibitors.

**Table 3:** Comparison of responders and non-responders, for age, sex, spondyloarthritis diagnosis and pharmacological treatment. Legend: Both responders and non-responders may have received more than one diagnosis or pharmacological treatment during the respective time frame

	Responders (*n* = 2785)^a^	Non-responders (*n* = 2986)^a^	*p*-value
Demographics			
Age, median (Q1, Q3)	57 (45, 66)	53 (41, 66)	<0.001
Sex, n women (%)	1494 (49)	1551 (51)	0.290
Diagnoses, *n* (%)			
Ankylosing spondylitis	744 (27)	679 (23)	0.001
Psoriatic arthritis	1285 (46)	944 (32)	<0.001
Undifferentiated SpA	411 (15)	300 (10)	<0.001
Sacroiliitis	157 (6)	209 (7)	0.035
Reactive arthritis	359 (13)	924 (31)	<0.001
IBD-associated arthritis	66 (2)	65 (2)	0.659
Spine enthesiopathy	13 (1)	12 (0)	0.842
Psoriatic spondylitis	20 (1)	19 (1)	0.750
Treatment, *n* (%)			
Methotrexate	936 (34)	538 (18)	<0.001
Sulphasalazine	297 (11)	159 (5)	<0.001
Etanercept	323 (12)	153 (5)	<0.001
Adalimumab	168 (6)	94 (3)	<0.001

Based on 5771 spondyloarthritis patients in the Skåne Health Care Register who were invited to participate in a survey. All data in the table is based on the health care registers

*Abbreviations*: *SpA* spondyloarthritis, *IBD* inflammatory bowel disease

^a^40 subjects had incomplete data and are excluded. Comparisons were performed using independent-sample t-tests and Fisher’s exact test

To indirectly examine the reliability of the survey and registry-derived data, kappa-values for variables that were captured by both data sources were calculated. The kappa-values (proportion of agreement in parenthesis) for SpA disease manifestations and current treatment were IBD, k = 0.65 (95 %); psoriasis, k = 0.70 (85 %); methotrexate, k = 0.87 (94 %); sulphasalazine, k = 0.91 (97 %); etanercept, k = 0.95 (98 %); and adalimumab, k = 0.95 (99 %).

## Discussion

In this population-based study the frequencies of current IBP were high in all three groups with AS, PsA and other-SpA. The proportion of patients afflicted with IBP was higher for women, compared to men, in PsA and other-SpA, but equal in AS, and the proportion of cases reporting current IBP was highest in AS, yet only 43 %. The three groups with current IBP had similar levels of self-perceived health status, reflecting pain, disease activity, function, and quality of life, which support the validity of IBP in non-AS SpA groups. However, there was a consistent trend for worse reports in the PsA and other-SpA groups compared to AS.

In analogy to our study, others have reported that AS and non-radiographic axial SpA patients attending rheumatology units have opposite gender distributions [2, 20, 21]. However, it should be stressed that we are comparing patients with different subtypes of clinically diagnosed SpA reporting current IBP, and not axial SpA according to the ASAS classification criteria, making comparisons to other studies difficult. The observation that AS and other axial SpA have similar health status is supported by a study of consecutive axial SpA patients at five rheumatology clinics in Germany, where compared with AS, the non-radiographic axial SpA phenotype was associated with equivalent self-reported health status [22]. One possible explanation for the relatively poor health status in PsA and other-SpA could be the female predominance in our groups with IBP in combination with PsA and other-SpA, since it is known that women in general report worse health status than men [23–26]. However, this notion is not supported by our analyses stratified by sex, were the trends observed for the PROMs in the gender-mixed groups were similar for both men and women separately (Additional file 1: Table S2). Another possible explanation for the poorer health status of these two groups could be insufficient treatment. However, our study showed that there were no statistically significant differences in pharmacological treatment between the AS and the “other-SpA” group and that the PsA-group was treated more frequently than the AS group, although non-pharmacological treatment was not accounted for. Since both psoriasis, with and without arthritis, as well as IBD are indications for treatment with sDMARDs and TNFi in Sweden [27, 28], the relatively high frequency of pharmacological treatment in these groups could be related to concurrence of psoriasis, IBD or peripheral arthritis. On the other hand, the similarly elevated mean levels of spinal pain, in the AS and non-AS SpA subgroups with current IBP may support the validity of IBP and presence of axial disease in these groups. In line with our results, a recent study based on the NHANES cohort found that there was a high frequency (17 %) of IBP in a group with “self-reported medically diagnosed psoriasis” [11]. The fact that only 43 % of patients with AS hade current IBP may be explained by the fluctuating course of symptoms, treatment effects and possibly due to decreasing symptoms with higher age.

Some limitations of the study must be discussed. First, the study setting relied on data from a postal survey and health care registers, which meant that we could not retrieve the physicians’ expert opinions regarding the existence of axial disease (i.e.*,* the “golden standard”). Second, the setting and study design also precluded assessment of biomarkers or imaging, which are central to both the modified New York criteria for AS and the ASAS criteria for axial SpA. However, validation studies based on a review of the clinical records in our setting have demonstrated a high validity for the diagnosis of AS on the basis of ICD-10 codes [12, 29]: over 80 % fulfilled the modified New York criteria, while 89 % fulfilled one or more of the criteria that are commonly used to classify patients with SpA. In this validation process, we also found that over 90 % of cases identified as undifferentiated SpA fulfilled at least one of the commonly used classification criteria of SpA, while only a minority fulfilled the modified New York criteria for AS [29]. Furthermore, a validation study of the ICD-10 codes for psoriasis in the cohort of the present study yielded positive predictive values [PPV] of at least 81 % [16]. In our study, the frequencies observed for anterior uveitis, psoriasis, and IBD in the AS group were also similar to those reported in other studies of radiographic axial SpA [20], also supporting the validity of the diagnoses. Based on these validation exercises, and the frequencies of SpA-related disease manifestations, we believe that the clinical diagnoses of SpA analyzed in this study are valid. Third, the high rate of non-responders may have affected the results, especially for the group with other-SpA. The lower frequencies of treatment with sDMARD and TNFi among non-responders could indicate lower rates of symptoms in this group, which has to be taken into consideration when trying to generalize our results.

The present study also has several strengths. It is one of the first population-based studies to assess and compare the frequency of current IBP within different SpA subtypes and to compare health status. Second, the information was gathered from several different sources. In particular, information regarding pharmacological treatment was gathered from a completely independent data source. Moreover, analysis of the reliability of our findings by comparing the data from the survey and the registers yielded kappa statistics that indicated a “good” reliability for IBD and psoriasis (k = 0.65 and 0.70, respectively) and a “very good” reliability for the pharmacological treatments (k = 0.87–0.95)[30].

## Conclusions

To sum up, our results suggest that the frequency of patients with current IBP is high in AS, PsA and other SpA, although the proportion is highest for AS. The impact on health status is, however, similar for patients with AS, PsA and other SpA with current IBP supporting the validity of this symptom in non-AS SpA groups. Our data also indicate that in our setting, this may already largely being recognized by the healthcare services; given that the groups with PsA and other-SpA received relevant pharmacological treatments at least as frequently as the AS group. However, there also appears to be room for improvement concerning pharmacological and non-pharmacological treatment, since a high proportion of cases still had symptoms and levels of PROMs suggesting an active axial disease, especially in the group with PsA.

## Additional files

Additional file 1: Supplementary **Table S1** and **S2**. Description of ICD-and ATC-codes used in the study and results stratified by sex. Supplementary Table S1 The ICD-10 and ATC-codes used to identify cases, spondyloarthritis related disease manifestations, and pharmacological treatment. Supplementary Table S2 The mean patient-reported outcome measures (PROMs) stratified by sex (DOCX 21 kb)

## Abbreviations

AS: ankylosing spondylitis
ASAS: assessment of spondyloarthritis
ATC: anatomical therapeutic code
BASDAI: Bath Ankylosing Spondylitis Activity Index
BASFI: Bath Ankylosing Spondylitis Functionality Index
EQ-5D: European Quality of Life-5 Dimensions index
IBD: inflammatory bowel disease
IBP: inflammatory back pain
ICD-10: international classification of diseases
NRS: numerical rating scale
NSAID: non-steroidal anti-inflammatory drug
PIN: personal identification number
PROM: patient-reported outcome measure
PsA: psoriatic arthritis
ReA: reactive arthritis
sDMARD: synthetic disease modifying drug
SHCR: Skåne Health Care Register
SpA: spondyloarthritis
TNFi: tumor necrosis factor alfa inhibitor
