# Supplementary material for: Back pain and health status in patients with clinically diagnosed ankylosing spondylitis, psoriatic arthritis and other spondyloarthritis: a cross-sectional population-based study
Source: BMC Musculoskelet Disord. 2016 Feb 27;17:106. doi: 10.1186/s12891-016-0960-8 (PMC4769824; doi:10.1186/s12891-016-0960-8)
Supplement: Additional file 1: — Supplementary Table S1 and S2. Description of ICD-and ATC-codes used in the study and results stratified by sex. Supplementary Table S1 The ICD-10 and ATC-codes used to identify cases, spondyloarthritis related disease manifestations, and pharmacological treatment. Supplementary Table S2 The mean patient-reported outcome measures (PROMs) stratified by sex (DOCX 21 kb) [file 12891_2016_960_MOESM1_ESM.docx]

| **SpA phenotypes** | **ICD-10 Code** |
| --- | --- |
| Ankylosing spondylitis | M45, M08.1 |
| Reactive arthritis | M01.2, M02.0-2, M02.8-9, M03.2 |
| Undifferentiated SpA | M46.8, M46.9 |
| Spine enthesiopathy | M46.0 |
| Sacroiliitis | M46.1 |
| Psoriatic arthritis | L40.5, M07.0-M07.3 |
| Spondyloarthritis associated with IBD  didiseaedisesDisease | M07.4-M07.5 |
|  |  |
| **SpA-related disease manifestations** |  |
| Inflammatory bowel disease | K50-K51 |
| Anterior uveitis | H20, H22.1 |
| Psoriasis | L40 |
|  |  |
| **ATC-codes for pharmacological treatment** | **ATC-codes** |
| Sulphasalazine | A07EC01 |
| Methotrexate | L01BA01, L04AX03 |
| Etanercept | L04AB01 |
| Adalimumab | L04AB04 |
| Abbreviations: SpA=spondyloarthritis, IBD= inflammatory bowel disease. | |

**Supplementary Table S1.**

The ICD-10 and ATC-codes used to identify cases, spondyloarthritis related disease manifestations, and pharmacological treatment.

**Supplementary Table S2** The mean patient-reported outcome measures (PROMs) stratified by sex.

| **PROMs:** | **AS with current IBP^1^ (n=319)** | **PsA with current IBP^1^ (n=409)** | **p-value^2^** | **Other-SpA with current IBP^1^ (n=282)** | **p-value^3^** | **p-value^4^** |
| --- | --- | --- | --- | --- | --- | --- |
| **Women, n (%)** | 137 (43) | 280 (68) |  | 195 (69) |  |  |
| NRS-spinal pain, mean (95% Cl) | 6.17 (5.82-6.52) | 6.27 (6.02-6.52) | 0.649 | 6.33 (6.05-6.62) | 0.480 | 0.748 |
| NRS-fatigue, mean (95% Cl) | 6.30 (5.90-6.70) | 6.33 (6.07-6.60) | 0.890 | 6.26 (5.90-6.62) | 0.876 | 0.733 |
| NRS-patients global, mean (95% Cl)^5^ | 5.42 (5.07-5.77) | 5.46 (5.21-5.71) | 0.840 | 5.38 (4.89-5.87) | 0.749 | 0.864 |
| BASDAI, mean (95% Cl) | 5.43 (5.07-5.78) | 5.75 (5.52-5.99) | 0.122 | 5.64 (5.34-5.93) | 0.363 | 0.547 |
| BASFI, mean (95% Cl) | 4.69 (4.27-5.11) | 5.01 (4.72-5.31) | 0.218 | 4.60 (4.25-4.94) | 0.726 | 0.702 |
| EQ-5D, mean (95% Cl) | 0.68 (0.65-0.71) | 0.65 (0.63-0.68) | 0.131 | 0.66 (0.63-0.69) | 0.381 | 0.537 |
|  |  |  |  |  |  |  |
| **Men, n (%)** | 182 (57) | 129 (32) |  | 87 (31) |  |  |
| NRS-spinal pain axial, mean (95% Cl) | 5.63 (5.29-5.96) | 5.91 (5.51-6.32) | 0.287 | 5.99 (5.54-6.44) | 0.221 | 0.807 |
| NRS-fatigue, mean (95% Cl) | 5.43 (5.10-5.77) | 5.78 (5.37-6.18) | 0.193 | 5.82 (5.28-6.36) | 0.213 | 0.902 |
| NRS-global, mean (95% Cl) | 4.94 (4.61-5.27) | 5.13 (4.76-5.50) | 0.446 | 5.38 (4.89-5.87) | 0.138 | 0.418 |
| BASDAI, mean (95% Cl) | 4.78 (4.47-5.10) | 5.41 (5.03-5.78) | 0.013 | 5.33 (4.87-5,79) | 0.053 | 0.806 |
| BASFI, mean (95% Cl) | 4.25 (3.86-4.64) | 4.22 (3.79-4.65) | 0.930 | 4.06 (3.50-4.63) | 0.593 | 0.651 |
| EQ-5D, mean (95% Cl) | 0.68 (0.65-0.71) | 0.66 (0.62-0.69) | 0.308 | 0.67 (0.63-0.72) | 0.743 | 0.595 |
| The data are derived from 2785 patients with AS or SpA in the Skåne Health Care Registry who responded to a questionnaire.  1) Current IBP: reporting ≥3 months of back pain in the preceding year and fulfilling the Berlin criteria for inflammatory back pain. 2, 3) AS *vs*. PsA, AS *vs.* Other SpA. 4) PsA *vs.* Other-SpA 5) Patient’s global assessment of back disease.  Abbreviations: NRS= Numerical Rating Scale 1–10; BASDAI= Bath Ankylosing Spondylitis Activity Index; BASFI= Bath Ankylosing Spondylitis Functional Index; EQ-5D= European Quality of Life-5 Dimensions; PROMs= Patient Reported Outcome Measures. | | | | | | |
